# HSV-1 Encephalitis: High Index of Clinical Suspicion, Prompt Diagnosis, and Early Therapeutic Intervention Are the Triptych of Success—Report of Two Cases and Comprehensive Review of the Literature

**DOI:** 10.1155/2017/5320839

**Published:** 2017-08-16

**Authors:** Dimitrios Patoulias, Georgios Gavriiloglou, Konstantinos Kontotasios, Mairi Tzakri, Petros Keryttopoulos, Christos Koutras

**Affiliations:** Department of Internal Medicine, General Hospital of Veria, Veria, Greece

## Abstract

Herpes Simplex Virus (HSV) encephalitis is an acute infectious disease of the Central Nervous System (CNS), usually affecting the limbic structures, the median temporal cortex, and the orbitofrontal regions. Its annual incidence has significantly increased over the last 20 years and the mortality rate is 7%, if early diagnosed and treated, and 70%, if left untreated, while it is associated with high rates of morbidity. It should be noted that even when Cerebrospinal fluid (CSF) analysis seems normal, imaging studies are not specific and HSV Polymerase Chain Reaction (PCR) test is negative; the clinician should be more aggressive, if clinical presentation is indicative for HSV encephalitis, by administrating acyclovir early after patient's admission. The latter may be a vital intervention for the patient, modifying the patient's clinical course. Through the presentation of two cases of HSV-1 encephalitis that we managed in our department over the last 1 year and after systematic and comprehensive research of the relevant literature, we aim at showing the crucial role of medical history and physical examination, along with the high index of clinical suspicion, in order to make promptly the diagnosis and administer timely intravenous acyclovir, limiting the possibility of complications during the disease's course.

## 1. Introduction

HSV encephalitis is acute necrotizing mesocortical and allocortical encephalitis. Its annual incidence is estimated up to 1.2/100.000/year, with a tenfold increase noticed during the last 20 years, in comparison with reports from the '90s [1, 2].

This acute infectious encephalopathy is associated with high morbidity and mortality. If left untreated, mortality rate exceeds 70%. Only in England, more than 700 cases of viral encephalitis occur yearly, with mortality rate about 7%.

In the large multicenter study by Granerod et al., 42% of all cases of encephalitis were infectious, while HSV was the main pathogen (19%), followed by VZV (5%) and* Mycobacterium tuberculosis* (5%) [3].

As for the increase in acute infectious encephalitis incidence, it seems that advance in diagnostic procedure, along with increase in identified causative pathogens—climate change and international travel raise are substantial—plays a significant role in this noticed increase. It should be noted that encephalitis remains an underreported disease [4].

Regarding epidemiology of HSV encephalitis in Greece, there are no recent published data. Based upon the latest published study in immunocompetent adult population, etiology of acute encephalitis was identified only in 18% of all included patients (*n* = 45), with Enteroviruses (9%) and HSV-1 (9%) being the most common identified pathogens [5].

An emerging cause of acute encephalitis in Greece is West Nile Virus. During 2010, an outbreak of WNV infection was documented in Greece. A total of 262 patients were infected by WNV, while neuroinvasive disease occurred in 197 of them. Death was documented in 35 patients. During the following years, 75, 109, and 51 cases of neuroinvasive disease due to WNV were reported in 2011, 2012, and 2013, respectively [6]. Nine, 18, and 11 patients died due to the disease, respectively.

## 2. Aim

We aim, through the presentation of two cases of HSV-1 encephalitis that we managed in our department during the last year and after systematic and comprehensive research of the relevant literature, at highlighting the undeniable role of high index of clinical suspicion regarding the diagnosis of this entity and the prompt therapeutic intervention. In highly suspected cases, and even without definite paraclinical documentation, clinician must be aggressive, by administering early after the onset of the symptoms acyclovir to the suffering patient.

## 3. Case 1

A 76-year-old female presented to the Emergency Department of the hospital with fever (up to 38.2°C) over the past 5 days. The patient was somnolent and complained of cough and nausea the past 2 days. Earlier that day the patient was examined by a private neurologist, who recommended brain CT scan. Brain CT scan of the patient was unremarkable for acute changes. Her medical history included only dyslipidemia. The last 24 hours before admission the patient was already receiving antibiotic treatment with amoxicillin/clavulanate and azithromycin, prescribed by a private General Internist.

Regarding clinical examination, the patient had anomic aphasia and sentence repetition, was disoriented in time and space and somnolent, and had difficulty in cooperating with the doctor's orders. Absence of neck stiffness (Kerning and Brudzinski signs negative) was documented. Except the crackling right lung sounds her review of systems was normal. Her vital signs were BP: 150/80 mmHg, temperature: 37.8°C, SpO_2_: 94%, and bpm: 77. Due to blood test results WBC: 5150, NEU%: 84, and CRP: 0.03 (the rest were within normal limits), the clinical presentation, and the physical examination, a lumbar puncture (LP) was ordered. The CSF results were as follows: 75 cells/mm^3^, with lymphocytic predominance (94%), Glu: 77 mg/dl (normal range 40–75), TP: 53.8 mg/dl (normal range 15–45), and LDH: 45 IU/l. Due to the high suspicion of viral encephalitis, we decided to choose the admission of the patient. Intravenous administration of acyclovir 10 mg/kg/8 h was the first therapeutic intervention, while Levetiracetam 500 mg × 2 was also administered as prophylactic treatment.

**Figure 1:**
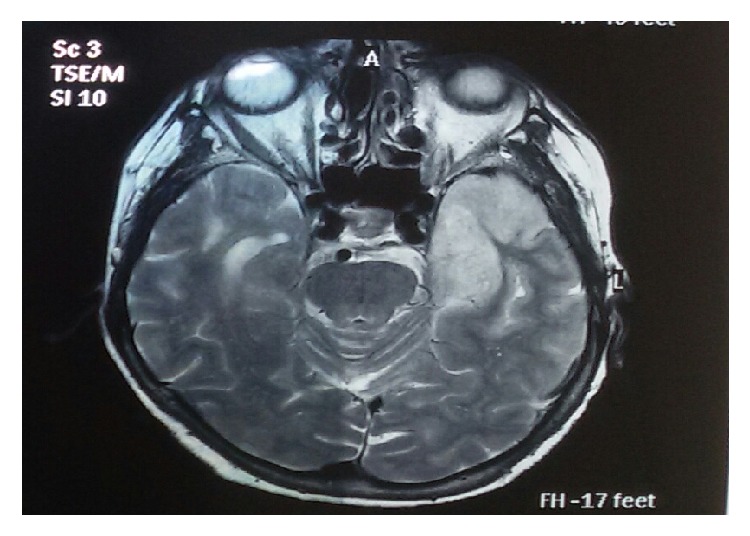
Hyperintense depiction of the left temporal lobe and the insula in T2WI sequence of brain MRI scan, indicative for HSV encephalitis.

Due to the possible diagnosis of viral encephalitis, we ordered soon after admission a brain MRI scan. The latter showed hyperintensity in the left temporal lobe and the insula in T2WI and FLAIR sequence, indicative for HSV encephalitis (Figure 1). CSF samples were collected and sent for HSV types I-II, EBV, VZV, and CMV examination and serum samples for WNV examination. Blood cultures (during episodes of fever) were also collected. The blood cultures were negative, the serum sample was negative for West Nile Virus (WNV), and the CSF sample was positive for HSV type I (2733 copies/ml) and negative for HSV-2, EBV, CMV, VZV, and Enteroviruses, leading to a definite diagnosis of HSV-1 encephalitis. Serology for HBV, HCV, and HIV was also negative. VDRL and RPR turned out negative, as well.

Over the next days of her hospitalization the patient remained stable, with normal blood test results and gradual improvement of her somnolence and aphasia. The patient was discharged home in excellent general condition, after completion of 14 days of intravenous administration of acyclovir. There are no complications reported so far, and the patient remains asymptomatic.

## 4. Case 2

A 56-year-old woman presented to the Emergency Department due to fever and mental status alteration over the past one week. Her medical history was unremarkable. The patient, after returning from her summer vacation, developed low fever, up to 37,8°C, while she was also complaining of mild frontal headache. Then, she visited a General Internist who treated her conservatively as a viral infection. Two days later, nausea and vomiting started. Fever rose up to 39°C and her mental status changed dramatically. She became somnolent, while in the last 3 days before hospital admission she became aphasic.

**Figure 2:**
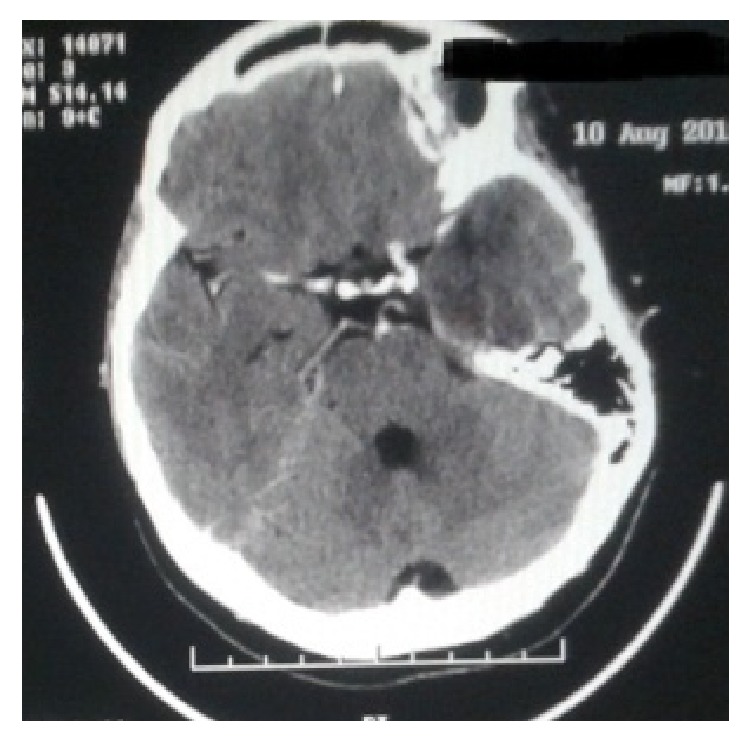
CT brain scan. Hypodense depiction of the left temporal lobe.

**Figure 3:**
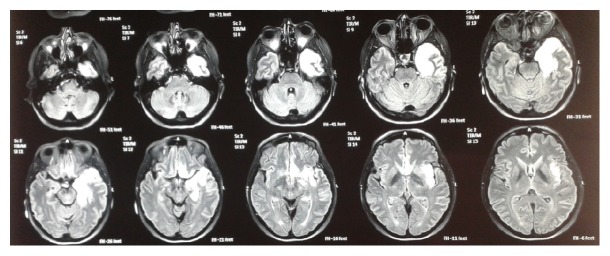
MRI brain scan. Notice the hyperintense depiction of the left temporal and the frontal lobe and the island of Reil in T2WI and FLAIR sequence.

At admission she was febrile (body temperature 38,1°C), while the rest of her vital signs were within normal limits. During the neurological examination she was alert, and she could obey simple orders but could not answer questions and form sentences. The upper and lower limbs showed normal muscular force and normal tendon reflexes. She suffered from central facial nerve paralysis on the right while she did not have neck stiffness and other meningeal signs. The rest of the clinical examination was normal. From the laboratory test results, low Na^+^ concentration was noticed (131 mmol/l). The rest was within normal range. The chest X-ray was normal and the brain CT scan showed hypodensity on the left temporal lobe (Figure 2). A LP was also performed. The CSF results were as follows: 364 cells/mm^3^ with lymphocytic predominance (99%), Glu 55 mg/dl, and TP 112.7 mg/dl. The patient was then admitted to the hospital with the suspicion of central nervous system infection. On second day of hospitalization a brain MRI scan was ordered, which showed hyperintense depiction of the left temporal and the frontal lobe and the island of Reil in T2WI and FLAIR sequence (Figure 3). CSF was also sent for PCR analysis for HSV types I and II, WNV, EBV, VZV, CMV, and Enteroviruses. The results were positive for HSV type I (12550 copies/ml), while being negative for the other viruses. Blood cultures (during episodes of fever) were also collected. They were all negative. Serology for HBV, HCV, and HIV was also negative. VDRL and RPR turned out negative, as well.

Therapeutic regimen of the patient included acyclovir 10 mg/kg/8 h, Levetiracetam, and dexamethasone. From the third day of hospitalization her aphasia began to resolve, and she was oriented to time and space, but the fever and the mild frontal headaches persisted for a week. She was discharged home in excellent general condition, after completion of 14 days of intravenous administration of acyclovir and with prescription for antiepileptic treatment as well.

## 5. Discussion

Clinical manifestations, imaging studies, and CSF analysis are the basis of diagnostic approach in encephalitis.

Fever, consciousness disorders, disorientation, behavioral disorders, language disorders, seizures or status epilepticus, and focal neurologic deficit constitute the typical signs and symptoms of HSV encephalitis [7]. Based upon the clinical presentation, both of our patients were highly suspected for viral infection of the CNS. According to the established diagnostic criteria for encephalitis and encephalopathy of presumed infectious or autoimmune etiology, both of our patients met the major criterion and at least three minor criteria [8]. At least two seizures or status epilepticus instances, severe organ damage, or behavior alteration is usually indication for temporary admission to ICU [9].

Urgently performing a brain CT scan should be considered as necessary in suspected cases of HSV encephalitis, prior to lumbar puncture, when one or more of the following are present: evidence of obstructive raised intracranial pressure, Glasgow Coma Scale (GCS) < 13, fall in GCS > 2, focal neurological signs, abnormal posturing, papilloedema, and immunocompromise [10]. It is not the imaging modality of choice in suspected viral encephalitis. A normal brain CT scan cannot rule out the diagnosis. Due to the presence of focal neurological signs in both of our patients, we requested a brain CT scan early after the initial clinical evaluation. Although brain CT scan is normal in about half of all patients, depiction of pathology in the second patient may be due to the delay in admission.

Performing a lumbar puncture is usually the next diagnostic step during the approach of a patient with possible infectious encephalopathy. Typically, CSF analysis shows pleocytosis, elevated protein and erythrocyte levels, and normal glucose levels [9]. In both of our patients, CSF analysis was abnormal, indicative for possible viral encephalitis and warranting further diagnostic investigation.

HSV CSF PCR test is the gold standard method for the diagnosis of HSV encephalitis, a test with 98% specificity and 94% sensitivity [11]. CSF sampling early or late after the onset of symptoms (<3 and >14 days, resp.) may reduce the possibility of a positive HSV PCR result [12].

At this point, we should note that first-line CSF PCR testing in highly suspected for encephalitis cases in immunocompetent patients includes the following pathogens: HSV 1/2, VZV, and Enteroviruses [3, 8]. However, both of our patients featured practically nonremarkable medical history and there was no recent or past hospitalization. Thus, we could not evaluate at the time of their admission their immunocompetency. The latter, along with the acute onset of symptomatology and the presence of clear neurological signs, led us to order initially a broader PCR testing, including pathogens that are causative factors of acute encephalitis in immunocompromised patients, such as EBV and CMV.

According to the study conducted by Ziyaeyan et al. regarding HSV PCR testing in CSF samples, the authors conclude the following: (1) the greater the initial viral load is, the longer the PCR test remains positive during the course of therapeutic intervention, (2) if CSF samples are collected early after the onset of the symptoms, PCR testing may turn out negative, (3) in cases of a negative first result, the clinician should repeat the test after two days, and (4) duration of acyclovir treatment less than 8 days does not usually turn PCR test to be negative [13]. In both of our cases, patients had symptomatology for 5 and 7 days, respectively, before admission, thus reducing the risk of a “normal” CSF sampling with negative PCR results, in the context of viral encephalitis.

In their study, Saraya et al. found that 6/23 patients (26.1%) with confirmed HSV encephalitis had normal WBC in collected CSF samples. Viral load in the pleocytosis group was higher than that in the normocellular group. Viral load did not affect the final clinical outcome [14]. In their retrospective study, Sheybani et al. documented that CSF analysis was normal in 18% of all patients with identified HSV encephalitis, while CSF HSV-1 PCR test was negative in 24% of all patients [15].

Evaluation of intrathecal synthesis of HSV specific IgG antibodies in the CSF, in combination with HSV PCR test, improves sensitivity of HSV detection in suspected cases [16]. In their cohort, Ambrose et al. document that antibody assessment in the CSF of patients with acute encephalitis was more probable to be positive at 14 to 28 days after admission than at 0 to 6 days after admission. They also report that those patients with positive HSV PCR testing were negative for oligoclonal intrathecal antibodies, while patients with undetected HSV DNA in CSF analysis featured positive intrathecal antibodies. Thus, they conclude that these two tests are complementary and useful for determination of different stages of the disease's course [17]. These antibodies remain positive in the CSF for several months or even years. In our case, we did not repeat a LP and thus we did not request HSV specific IgG antibodies detection.

Most usual radiologic features in HSV encephalitis are as follows: (1) hypodense lesions of temporal lobes and orbitofrontal regions, sometimes with petechial hemorrhage on brain CT scanning, and (2) hypointensity in T1 and hyperintensity in T2 images on brain MRI scanning. Brain MRI scanning was compatible with HSV encephalitis in both of our patients, affirming our high index of clinical suspicion and our choice to administer early after admission acyclovir intravenously, without waiting for the PCR test results [18].

According to the study conducted by Chow et al. in patients with manifestations of encephalitis and temporal lobe abnormalities on MRI scan, the presence of bilateral temporal lobe involvement and lesions outside the temporal lobe, insula, or cingulate were less frequently seen in patients with HSV encephalitis (*p* = 0.01 and 0.005 resp.). Mesial temporal sclerosis, gliomatosis cerebri, and MELAS should be included in differential diagnosis, when bilateral temporal lobe involvement is detected on MRI scan [19]. Paraneoplastic limbic encephalopathy, Hurst's disease, SLE, primary CNS lymphoma, complex partial status epilepticus, and neurosyphilis should also be included [20]. Temporal lobe involvement was unilateral in both of our patients.

Sheybani et al. found that brain CT scanning was abnormal only in 50% of the study group, while brain MRI was indicative of encephalitis in 92% of all patients. In accordance with the abovementioned regarding CSF biology, the authors suggest that even when CSF analysis and imaging studies are not suggestive of the diagnosis, diagnosis of HSV encephalitis cannot be excluded, when high index of clinical suspicion is present. Thus, in cases of febrile encephalopathy of unknown origin, the early administration of acyclovir may be crucial for the patient's survival [15]. Saraya et al. reached a similar conclusion, after noticing that 4 out of 6 patients with normal CSF analysis featured a nonspecific brain MRI scan and PCR testing was not available at the time of diagnosis [14].

According to the study performed by Poissy et al., chronic alcohol abuse, high Knaus score, and delay in the first brain CT or MRI scan after admission were the three factors associated independently with delay in administration of acyclovir (*p* = 0.02, 0.007, and <0.001, resp.). White cell count < 10/mm^3^ in CSF increased 2.5-fold the risk of late administration of acyclovir, although that finding was not found to be statistically significant (*p* = 0.17). When the clinician confronts a patient with severe underlying disease or chronic alcohol abuse, in high index of suspicion for HSV encephalitis, even when CSF appears normal, early initiation of acyclovir is crucial for the final outcome, based on the fact that delay in acyclovir administration is one of the most important modifiable prognostic factors [21].

In their retrospective study, Wang and Liu emphasize the fact that MRI imaging may be of higher sensitivity for the diagnosis of HSV encephalitis, compared with HSV PCR test. Based on the fact that HSV PCR test may turn out negative early after the onset of the clinical manifestations, the authors suggest the prompt conduction of MRI and early administration of acyclovir, in cases highly suspected for encephalitis. Thus, MRI is crucial for the appropriate therapeutic intervention, in accordance with the clinical presentation, and sole reliance on PCR test should be avoided [22]. Due to that fact, we ordered the conduction of a brain MRI scan on the second day of hospitalization in both patients, having already started acyclovir administration and without waiting for PCR test results.

Regarding prognosis, male gender, increasing age, lower GCS score, and delay in initiation of acyclovir >2 days after the onset of the symptoms are the main predictors of unfavorable outcome in HSV encephalitis, according to Erdem et al. [23]. In their retrospective study in patients with acute encephalitis, Thakur et al. [24] found that mortality was 29% higher in the presence of cerebral edema identified radiologically (*p* < 0.01), 21% higher when status epilepticus occurred (*p* = 0.01), and 19% higher when thrombocytopenia developed (*p* = 0.01) during the disease's course. Patients > 65 years old, immunocompromised, or/and with comorbidities were at higher risk of death, a finding that is not statistically significant. None of our patients had clinical or radiologic signs of cerebral edema, developed status epilepticus, or featured thrombocytopenia during their hospitalization.

## 6. Conclusion

Despite the fact that the two cases we presented were typical, regarding clinical presentation and paraclinical examination, we believe that the diagnostic and therapeutic strategy of the clinician should be based upon the parameter of economy of time, as there is absolute indication for early administration of acyclovir in highly suspected cases. However, even when CSF analysis is within normal limits, imaging studies are nonspecific and PCR test is negative, and diagnosis of HSV encephalitis could not be ruled out, if clinical presentation is highly indicative. Thus, high index of clinical suspicion, prompt diagnosis, and early therapeutic intervention are the triptych of success.

## Abbreviations

HSV: Herpes Simplex Virus
LP: Lumbar puncture
CSF: Cerebrospinal fluid
CT: Computed Tomography
MRI: Magnetic Resonance Imaging
PCR: Polymerase Chain Reaction
GCS: Glasgow Coma Scale.
